# Flexible Bioelectrodes-Integrated Miniaturized System for Unconstrained ECG Monitoring

**DOI:** 10.3390/s25134213

**Published:** 2025-07-06

**Authors:** Yaoliang Zhan, Xue Wang, Jin Yang

**Affiliations:** 1 Chongqing Key Laboratory of Photo-Electronic Functional Materials and Laser Technology, College of Physics and Electronic Engineering, Chongqing Normal University, Chongqing 401331, China; 2022051110032@stu.cqnu.edu.cn; 2 Chongqing Municipal Key Laboratory of Photo-Electronic Materials and Engineering of Higher Education, College of Physics and Electronic Engineering, Chongqing Normal University, Chongqing 401331, China; 3 Key Laboratory of Optoelectronic Technology and Systems, Department of Optoelectronic Engineering, Ministry of Education, Chongqing University, Chongqing 400044, China

**Keywords:** wearable system, flexible bioelectrode, ECG monitoring

## Abstract

The electrocardiogram (ECG) signal plays a crucial role in medical diagnosis, home care, and exercise intensity assessment. However, traditional ECG monitoring devices are difficult to blend with users’ daily routines due to their lack of portability, poor wearability, and inconvenient electrode usage methods. Therefore, utilizing reusable and cost-effective flexible bioelectrodes (with a signal-to-noise ratio of 33 dB), a miniaturized wearable system (MWS) is proposed for unconstrained ECG monitoring, which holds a size of 65 × 52 × 12 mm^3^ and a weight of 69 g. Given these compelling features, this system enables reliable and high-quality ECG signal monitoring in individuals’ daily activities, including sitting, walking, and cycling, with the acquired signals exhibiting distinguishable QRS characteristics. Furthermore, an exercise intensity classification model was developed based on ECG characteristics and a fully connected neural network (FCNN) algorithm, with an evaluation accuracy of 98%. These results exhibit the promising potential of the MWS in tracking individuals’ physiological signals and assessing exercise intensity.

## 1. Introduction

As a fundamental tool for assessing cardiac function, the electrocardiogram (ECG) signal provides comprehensive insights into cardiac structure, functional state, and electrophysiological characteristics [1,2,3,4,5,6]. It is a critical reference in cardiac health monitoring, disease diagnosis, risk stratification, and telemedicine applications [7,8,9,10,11,12,13,14]. As individual health awareness continues to rise, there is a growing need for comfortable, high-fidelity ECG monitoring [15,16,17]. However, conventional ECG monitoring systems rely on external lead wires and fixed measurement configurations and often suffer from high cost, limited portability, and complex electrode fabrication processes. These challenges substantially hinder the widespread adoption of wearable health monitoring technologies. Recent advances in flexible sensing materials and integrated electronics have propelled the development of miniaturized wearable devices capable of unconstrained ECG signal acquisition. These emerging technologies are designed to meet the growing need for continuous, real-time health surveillance in everyday settings, enabling ubiquitous and seamless cardiac monitoring.

As a typical bioelectrical signal, the ECG signal is transmitted through direct contact between bioelectrodes and the skin surface [18,19,20]. Consequently, the flexibility and electrical conductivity of bioelectrodes are critical factors that affect the comfort and accuracy of ECG monitoring. Textile-based electrodes, renowned for their flexibility and wearing comfort, have been extensively employed as wearable biosensors for physiological signal monitoring [21,22,23,24]. For instance, Villar and co-workers validated the effectiveness of the Hexoskin smart vest during various activities, including lying, sitting, standing, and walking [25]. Hashimoto and co-workers developed a wearable heart rate monitor consisting of a smart shirt with integrated bioelectrodes and a wireless transmitter, verifying its accuracy in heart rate monitoring during light to moderate physical work [26]; Dong and co-workers successfully developed multifunctional textile-based bioelectrodes exhibiting high sensitivity, super-stretchability, self-cleaning ability, and strong environmental tolerance, significantly enhancing the robustness of bioelectrode performance under varying conditions [27]. However, textile electrodes inherently lack the ionic-to-electronic conversion mechanism that gel-based Ag/AgCl layers provide, resulting in high skin-electrode impedance and low contact signal-to-noise ratio (SNR) [28]. Moreover, insufficient micro-scale conformity of the textile surface to the skin interface exacerbates capacitive coupling losses and further degrades signal fidelity. Conversely, gel-based electrodes offer superior skin adhesion and favorable dielectric properties, making them promising candidates for reliable ECG monitoring [29,30,31,32,33]. For example, Le and co-workers introduced a novel ionogel material with exceptional stretchability, self-healing capability, biocompatibility, and long-term durability [34]. Moreover, extensive endeavors have been undertaken to explore a myriad of materials for fabricating bioelectrodes, aiming to improve signal quality and user comfort [35,36,37,38,39,40,41,42,43], thereby accelerating the advancement of wearable physiological signal monitoring technologies. Nevertheless, most conventional bioelectrode fabrication remains complex and costly, which hinders their scalability and practical deployment in portable monitoring systems. In addition, miniaturization of the device is equally vital to meet the demands of ECG monitoring. Although existing studies have made progress in either electrode optimization or system integration, most concentrate on a single aspect, with few providing a comprehensive solution that effectively integrates both. Therefore, it is essential to develop electrodes with high stability and biocompatibility while designing highly integrated signal conditioning circuits tailored to their electrical characteristics. This integrated approach is crucial for advancing the miniaturization of wearable ECG monitoring systems and enabling long-term, stable ECG signal acquisition. To address these challenges, we developed a highly moldable flexible bioelectrode (FBE) using food-grade materials through a simple, low-cost fabrication process. The self-fabricated FBE achieves a high signal-to-noise ratio (SNR) of 33 dB and demonstrates excellent reproducibility in ECG monitoring, validated by daily 100-s recordings over one month. Its superior moldability enables direct shape adaptation to the skin without the need for pre-formed molds, highlighting its potential for customized and conformal applications [44,45]. Building on this foundation, we further developed a Miniaturized Wearable System (MWS) incorporating the FBE for unconstrained ECG monitoring. Users can simply place both thumbs on the electrodes, and the system performs real-time signal acquisition, filtering, visualization, and data storage. Compared to commercial solutions such as AliveCor Kardia, the MWS offers significant advantages in compactness, lightweight design, and cost-effectiveness. In addition, leveraging the high-quality ECG signals acquired by the MWS, we trained a fully connected neural network (FCNN)-based model for exercise intensity classification. By extracting key ECG features, such as R-wave to R-wave (RR) interval, Q-wave to T-wave (QT) interval, and P-wave to R-wave (PR) interval, the model achieved 98% accuracy after 146 training epochs. These promising results position the MWS as a versatile and cost-effective platform for advancing innovation in portable ECG monitoring.

## 2. Experimental Section

### 2.1. Preparation of Materials

The required materials include 250 g of flour, 135 mL of NaCl solution, 10 g of tartaric acid (Henan Zhongchen Biotechnology Co., Ltd., Zhengzhou, Henan, China), and 8 mL of vegetable oil. First, tartaric acid is added to the NaCl solution, followed by the gradual addition of vegetable oil with continuous stirring for 1–3 min to ensure complete dissolution. The solution is then heated to 50 °C over low heat, and 200 g of flour is gradually incorporated. At this stage, vigorous and continuous stirring for 5–10 min is essential to achieve thorough mixing until a dough-like consistency is formed. The mixture is then left to rest at room temperature (20–25 °C) for 8–12 min. After cooling, it is transferred to a dry container, where 50 g of flour is gradually added and stirred vigorously for 5–8 min until the dough attains the desired viscosity. At this point, the FBE is fully prepared for physiological signal monitoring.

### 2.2. Impedance Testing

The electrode-skin interface impedance was measured using an impedance analyzer to ensure high-quality acquisition of physiological signals. The measurement procedure was as follows:

1. The volunteer’s skin was cleansed with 70% isopropyl alcohol and allowed to dry.
2. Two biopotential electrodes were positioned on the skin surface at a fixed inter-electrode distance.
3. The electrodes were connected to the impedance analyzer’s two terminals, forming a closed electrode-skin-electrode circuit.
4. The measurement was conducted using a frequency sweep range of 20 Hz to 15 kHz under an AC excitation voltage of 10 mV (rms). This excitation amplitude was carefully chosen to ensure a sufficiently small signal, thereby preserving the linearity of the system throughout the test. The impedance analyzer was then used to acquire the measurement data.

### 2.3. Humidity-Resistance Testing

The FBE was placed indoors (T=15 °C to 20 °C, RH=45% to 55%) and integrated into a resistive voltage divider circuit for resistance measurement. A temperature and humidity sensor was deployed to monitor the relative humidity in real-time, and data were recorded continuously for 12 h.

### 2.4. Composition of the MWS

The MWS is powered by a 3.7 V lithium battery, with its voltage regulated to 5 V using the LGS5500EP (Legend-Si, Shenzhen, China) voltage regulator to ensure stable operation of the filtering circuit. This regulator also integrates a charging management function, allowing for repeated use of the MWS. Additionally, the XC6206 (TOREX Semiconductor Ltd., Tokyo, Japan) chip functions as a low dropout (LDO) linear regulator, stepping down the voltage to 3.3 V to supply the microcontroller unit (MCU, STM32F407VGT6, STMicroelectronics, Geneva, Switzerland). A high-precision operational amplifier, the GS8552-SR (Gainsil Semiconductor Technology Co., Ltd., Shanghai, China), is incorporated into the filtering circuit, with a quiescent current of only 180 μA, meeting the system’s low-power consumption and extended operational requirements. Furthermore, the ECB02C (Shenzhen Yijia IoT Technology Co., Ltd., Shenzhen, China) integrated Bluetooth chip is selected due to its compact size and low power consumption, ensuring compliance with the system’s miniaturization requirement. The AD8232 (Analog Devices, Inc., Wilmington, MA, USA) chip is employed as the analog front-end, incorporating an instrumentation amplifier with a high input impedance of 10 GΩ and a common-mode rejection ratio (CMRR) of 80 dB over the DC to 60 Hz frequency range, effectively suppressing common-mode interference. In the peripheral circuit, the Driven Right Leg (DRL) output is connected to both hand electrodes via 10 MΩ pull-up resistors. Additionally, the RLD_FB pin forms an integrator circuit in combination with an internal 150 kΩ feedback resistor and an external 1 nF capacitor. This configuration establishes a low-frequency drive loop with a cutoff around a few hertz, which helps stabilize the electrode-body interface potential and further attenuate powerline interference.

## 3. Results and Discussion

### 3.1. The Design of the MWS for ECG Monitoring

**Figure 1:**
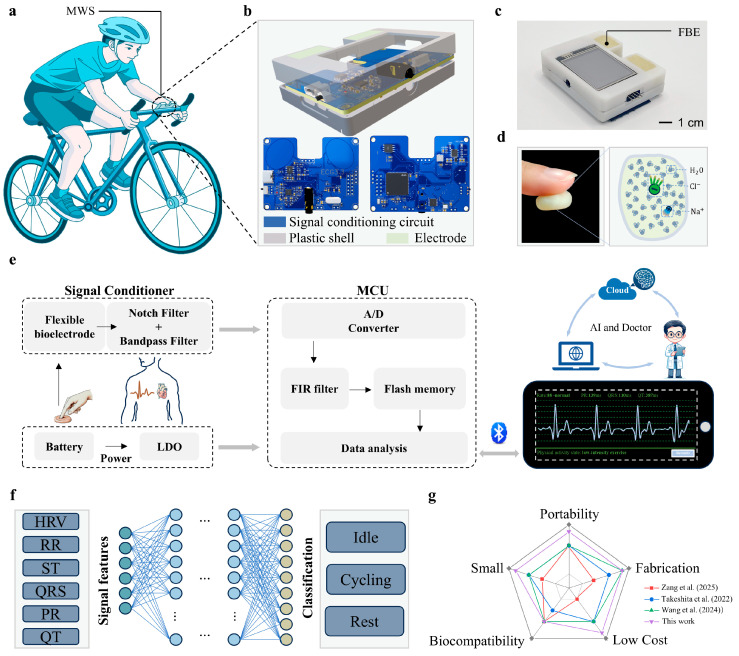
Wearable system for daily life ECG monitoring. (**a**) Schematic diagram illustrating the MWS worn on the human body or mounted on a bicycle handlebar. (**b**) Components of the MWS. (**c**) Physical image of the fully assembled MWS. (**d**) Schematic illustration of the adhesive properties of the FBE. (**e**) Signal processing workflow of the MWS for ECG signal analysis. (**f**) Structure overview of the model used for motion intensity classification. (**g**) Comparative analysis of this work with other related studies.

With the growing awareness of health, an increasing number of people are engaging in physical exercise to maintain fitness and relieve stress. Appropriate exercise intensity is essential for achieving the intended health benefits, as excessive or insufficient intensity may lead to adverse or ineffective outcomes. To enable real-time assessment of exercise intensity during daily workouts, we developed a miniaturized wearable physiological signal monitoring system, as shown in Figure 1a. The system supports multiple wearing configurations. It can be worn on the arm or mounted onto common exercise equipment (e.g., bicycle handlebars), facilitating versatile, scenario-adaptive use. During the monitoring process, users can achieve reliable ECG signal acquisition simply by placing their thumbs on the exposed electrode surfaces, which eliminates the need for external lead wires. Extracted ECG signal features are then used for automatic evaluation of exercise intensity. Compared to conventional ECG monitoring devices that rely on complex electrode wiring, the MWS streamlines the process with a “touch-and-measure” design, offering users enhanced freedom and convenience during monitoring. As illustrated in Figure 1b, the 3D model of the system integrates a signal conditioning circuit, a protective enclosure, and flexible bioelectrodes into a compact unit. With a dimension of 65 × 52 × 12 mm^3^ and a total weight of 69 g, the device ensures optimal portability for daily activities. Additional system details, including multi-view illustrations and physical interfaces, are provided in Figure S1 in the Electronic Supplementary Material (ESM).

In the design of the MWS, the FBE serves as a critical component, significantly influencing the quality of the acquired ECG signal. The FBE developed in this study is composed primarily of water and dough, exhibiting excellent environmental friendliness, non-toxicity, and high moldability, without the need for prefabricated shapes. Importantly, its hydration-induced adhesiveness provides suitable skin conformity (Figure 1d), which effectively reduces electrode-skin contact impedance and ensures stable signal acquisition. Benefiting from these properties, the FBE can be directly integrated into the system’s signal input ports without requiring additional electrical connection structures, thereby significantly enhancing the compactness and integration level of the device (Figure 1c). The raw ECG signal collected by the FBE is subsequently processed through a combination of hardware and software modules before being wirelessly transmitted to a smartphone application (APP), enabling real-time visualization of both ECG waveforms and characteristic physiological parameters. As illustrated in Figure 1e, the signal processing workflow begins with analog conditioning, including a 50 Hz notch filter (analog domain) to eliminate powerline interference and a bandpass filter to isolate relevant cardiac frequency components. The conditioned signals are then digitized by the analog-to-digital converter (ADC) integrated into the MCU. To further suppress residual noise and improve waveform clarity, a finite impulse response (FIR) digital filter is applied in software. The processed signals are subsequently transmitted via Bluetooth to the APP for real-time display and analysis. Thanks to its compact form factor and fully unconstrained measurement mode, the proposed system holds considerable potential for future integration with artificial intelligence algorithms, cloud computing platforms, and remote medical services, thereby enabling personalized health monitoring and rapid emergency response during physical activities.

By harnessing the high-quality ECG monitoring capability of the MWS, we further developed a deep learning-based model (Figure 1f) for exercise intensity classification. By extracting time-domain and frequency-domain features of the QRS, including heart rate variability (HRV), RR interval, ST interval, PR interval, QT interval, and QRS durations, we trained FCNN to distinguish among different levels of physical exertion and rest states during cycling. After 146 training epochs, the model achieved a classification accuracy of 98%, demonstrating its strong potential for real-time exercise assessment. The MWS exhibits comprehensive capabilities in ECG signal acquisition, conditioning, and intelligent analysis, showing distinct advantages over existing wearable systems. To quantify the advantages of the MWS, we compared it with other studies based on production cost, electrode material biocompatibility and fabrication simplicity, device size, and portability. The quantitative results are presented in Figure 1g. For instance, Zang and co-workers developed a contact-type heart sound sensor based on Pb(Zr,Ti)O_3_ (PZT) composite materials, achieving high SNR through sophisticated filtering techniques [46]. However, its relatively large physical footprint imposes limitations on long-term and portable monitoring. Takeshita and co-workers proposed a cubic flocked electrode by electrostatically depositing silver-coated fibers onto polyurethane substrates, offering excellent breathability and comfort [47], yet its intricate fabrication process constrains scalability. Similarly, Wang and co-workers’ silver-coated fiber/silicone (AgCF-S) dry electrode faces comparable challenges in terms of fabrication complexity [48]. In contrast, the MWS demonstrates superior performance with its exceptional portability, straightforward fabrication process (The detailed cost calculations are provided in Table S1), cost-effectiveness, and user-friendly operation. These advantages establish the MWS as a more practical and accessible solution for wearable ECG monitoring in real-world applications.

### 3.2. Preparation and Analysis of the FBE

**Figure 2:**
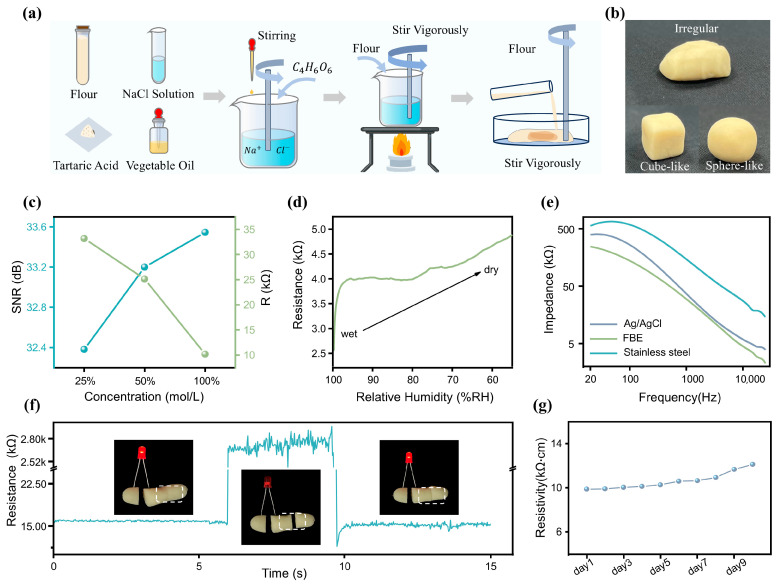
The FBE for ECG signal detection. (**a**) Fabrication process of the flexible FBE. (**b**) Demonstration of the FBE’s high plasticity. (**c**) SNR and resistance of the FBE prepared with NaCl solution at different concentrations under physiological signal monitoring conditions. (**d**) Correlation between FBE humidity and resistance. (**e**) Comparison of contact impedance among Ag/AgCl, stainless steel, and FBE on human skin with a contact area of 1 cm^2^. (**f**) Self-reshaping capability of the FBE. (**g**) Variations in the resistivity of the FBE over ten days.

The high-quality ECG signal acquisition in this system is attributed to the excellent conductivity of the FBE, which is composed of an electrolyte solution (NaCl solution), flour, tartaric acid, and vegetable oil. As illustrated in Figure 2a, the FBE is fabricated through a simple process of mixing and heating these components, with detailed preparation steps provided in the Section 2.

The outstanding material properties of the FBE are attributed to its protein constituents, mainly glutenin and gliadin, which form an interconnected network structure through hydrogen bonding, disulfide linkages, and hydrophobic interactions [49]. This unique molecular architecture imparts the dough with excellent moldability and natural adhesiveness. As depicted in Figure 2b, the FBE exhibits high plasticity and can be readily shaped into various forms, which is particularly advantageous for achieving seamless integration with physiological signal monitoring devices.

FBE, the electrolyte, ionizes into free anions and cations, whose movement imparts excellent conductivity to the dough. Therefore, the concentration of the NaCl solution has a significant impact on the conductive properties of the FBE. During the fabrication of the FBE, a series of optimization strategies were implemented to enhance its overall performance. As shown in Figure 2c, while maintaining consistent proportions of flour, tartaric acid, and vegetable oil, as well as processing time, we tested and compared the SNR of the FBE prepared with different concentrations of NaCl solution (100%, 50%, and 25% saturated solution) in physiological signal monitoring. Additionally, we measured the resistance across a rectangular FBE sample (a dimension of $3.5\times 1\times 1\;{cm}^{3}$) under these three conditions, to assess the effect of NaCl solution concentration on its conductivity. As shown in Figure 2c, experimental results revealed a clear positive correlation between the concentration of the NaCl solution and the performance of the bioelectrode. Higher concentrations led to markedly improved electrical conductivity, thereby enhancing the SNR during physiological signal acquisition. Among the tested formulations, the fully saturated NaCl solution demonstrated the best conductive properties, yielding the highest SNR. Based on these findings, this optimized composition was selected as the standard formulation for all subsequent experiments in this study. In addition, the moisture content of the FBE is a critical factor influencing its electrical conductivity. To investigate this, we conducted resistance measurements under dry environmental conditions using the setup shown in Figure S2 in the ESM. Keeping the size and shape of the FBE constant, we monitored its resistance as the relative humidity decreased from 100% to 55% due to moisture evaporation (Figure 2d). The results showed that the resistivity remained relatively stable within the 90% to 65% relative humidity range. To further quantify the electrical performance of the FBE, electrochemical impedance spectroscopy was performed using an impedance analyzer (model details provided in Table S2 in the ESM). Under the conditions of a cross-sectional area of 1 cm^2^ and a length of 2.5 cm, the impedance and phase response were measured across a frequency range of 20 Hz to 15 kHz (Figure S3 in the ESM). Notably, the relatively high impedance in the low-frequency range enables effective suppression of low-frequency noise, thereby improving the SNR. As a bioelectrode designed for direct skin contact, its skin-electrode contact impedance is of particular relevance. Therefore, we measured and compared the contact impedance of three types of electrodes: commercial Ag/AgCl gel electrodes, stainless-steel electrodes, and the FBE. All measurements were conducted with a contact area of 1 cm^2^ and across a frequency range from 20 Hz to 15 kHz, as illustrated in Figure 2e. Due to its lower surface moisture, the stainless-steel electrode exhibited significantly higher contact impedance than both the Ag/AgCl and FBE electrodes. Furthermore, the FBE showed even lower contact impedance than the Ag/AgCl electrode, indicating superior performance in physiological signal monitoring.

Notably, the interconnected protein network within the FBE exhibits a fascinating self-healing capability that allows it to rapidly restore electrical conductivity after mechanical damage. As shown in Figure 2f, we designed a simple circuit with an LED bulb connected across two FBE segments to visually assess conductivity recovery under an applied voltage. Upon reconnection of the fractured segments, the FBE instantly regained conductivity, illuminating the LED. This self-recovery property greatly enhances the reliability of the system during physiological signal monitoring, particularly in dynamic or mechanically demanding environments. In addition, we measured the resistivity changes in the FBE over 10 consecutive days under indoor conditions (Figure 2g). Due to moisture evaporation within the FBE, its resistivity slightly increased (Figure S4 in the ESM) but remained relatively stable overall, demonstrating its potential for long-term application.

### 3.3. Optimization of Signal Conditioning Circuit

**Figure 3:**
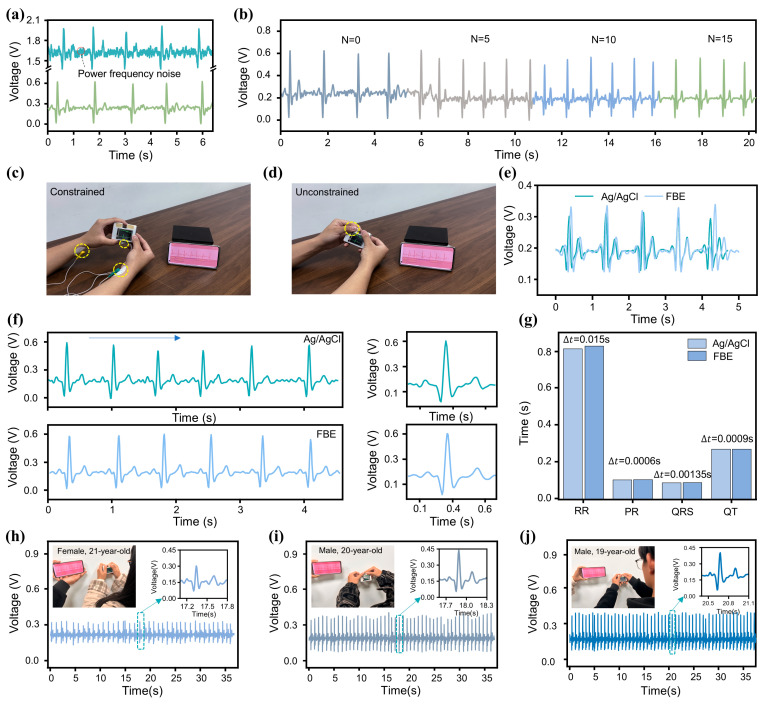
Optimization of MWS for ECG monitoring. (**a**) Waveforms of ECG signals before (top) and after (bottom) passing through the notch filter. (**b**) Waveforms of ECG signals processed by FIR filters with different orders. (**c**,**d**) Application of Ag/AgCl and FBE bioelectrodes for ECG signal measurement using the MWS. (**e**,**f**) ECG waveforms recorded with the two types of bioelectrodes. (**g**) Comparison of ECG signal feature values measured by the two electrodes. (**h**–**j**) ECG monitoring using the MWS on subjects of different genders and age groups.

During practical ECG monitoring, the acquired signal is highly susceptible to external noise interference, with 50 Hz powerline interference being particularly prominent. To ensure high-quality ECG signal acquisition, the design of an appropriate filter is critical. In this work, we implemented a Twin-T notch filter, with its circuit topology and frequency response characteristics presented in Figure S5 in the ESM. The active notch filter utilizes a twin-T network driven by two operational amplifiers within a negative feedback configuration to enhance frequency selectivity. The operational amplifiers effectively isolate the input and output of the twin-T network, eliminating mutual interference, while also providing the necessary gain for the feedback loop. This configuration significantly improves the notch depth and stopband performance. This filter effectively attenuates 50 Hz power frequency noise while preserving the integrity of other signal components, although slight attenuation occurs near the cutoff frequency. To evaluate the filter’s performance, we compared ECG waveforms before and after filtering. As shown in Figure 3a, the waveform processed by the Twin-T notch filter appears smoother, while key features of the original signal are well preserved, demonstrating the filter’s strong capability in suppressing 50 Hz noise. Additionally, we incorporated an FIR filter (a low-pass filter employs a Hamming window with a 50 Hz cutoff frequency) into the system. Figure 3b shows the filtering performance of the FIR filter with various orders (*N*). While higher-order filters offer superior noise suppression, they also impose a greater computational burden on the microcontroller and reduce the effective signal sampling rate. After balancing noise reduction efficiency with the microcontroller’s processing capacity, an FIR filter with order N=15 was ultimately selected (The detailed software architecture of the microcontroller can be found in Figure S6 in the ESM).

To validate the accuracy of the MWS as an ECG monitoring device, we conducted comparative tests against a standard commercial monitoring system. As shown in Figure S7a, simultaneous ECG recordings were obtained from a healthy subject using both the MWS and a standard multi-parameter patient monitor (PM12B, Chengyitong, Changsha Yinuowei Medical Technology, Changsha, China). The results, presented in Figure S7b, demonstrate that despite minor differences in waveform morphology, attributable to variations in lead configurations, the key characteristic waves exhibit strong temporal alignment. Quantitative analysis yielded a Pearson correlation coefficient of 0.74, indicating substantial signal consistency between the two systems. Furthermore, the mean RR interval difference was 0.03 s, and the mean PP interval difference was 0.02 s, further confirming the MWS’s reliability and accuracy in ECG monitoring. Additionally, to verify the accuracy of the FBE in capturing the ECG signal, we conducted short-interval comparative tests on the same subject using both commercial Ag/AgCl gel electrodes and the FBE. Figure 3c shows an individual undergoing ECG testing using commercial Ag/AgCl gel electrodes. The two electrode patches were attached to the wrists of both hands and connected to the MWS via wires. The individual’s ECG waveform is displayed on the screen (Video S1 in the ESM). In contrast, the FBE-based measurement setup (Figure 3d) required only that the subject place both hands on the exposed electrodes of the MWS to obtain accurate ECG waveforms (Video S2 in the ESM). The results presented in Figure 3e reveal a high degree of waveform similarity between the two electrode types, with minor differences attributed to natural HRV during the sequential recordings. For a more detailed signal quality comparison, Figure 3f shows the enlarged and single-cycle waveforms from both methods. Notably, the wired measurement using Ag/AgCl electrodes exhibited some powerline interference due to long wires, while the FBE-based unconstrained measurement showed no significant noise, highlighting its superior portability and practicality for ECG signal acquisition. Additionally, feature value analysis was performed to verify the completeness of the ECG signal captured by both electrodes (Figure 3g). The maximum feature value difference was t=0.015 s, demonstrating the FBE’s capability to accurately acquire the subject’s ECG signal.

To evaluate the adaptability of MWS across different individuals, we conducted 30-s tests on three subjects of varying ages and genders under identical conditions. As shown in Figure 3h–j, the single-cycle waveforms confirm that all characteristic waves were clearly and completely captured for each subject. Although the ECG signal waveforms exhibited some differences due to variations in physical conditions (such as emotional state, exercise habits, and age) among the three individuals, these differences are consistent with normal physiological variability, indicating that the MWS possesses a high degree of individual adaptability. To verify the long-term applicability of the MWS, 1-min ECG signal recordings were performed daily over one month in a humid environment. As illustrated in Figure S8 in the ESM, the ECG signals collected by the MWS exhibited no significant degradation except for a slight reduction in amplitude after one month of use, confirming its strong long-term stability and reliability under challenging environmental conditions.

### 3.4. ECG Monitoring Using the MWS in Different Scenarios

**Figure 4:**
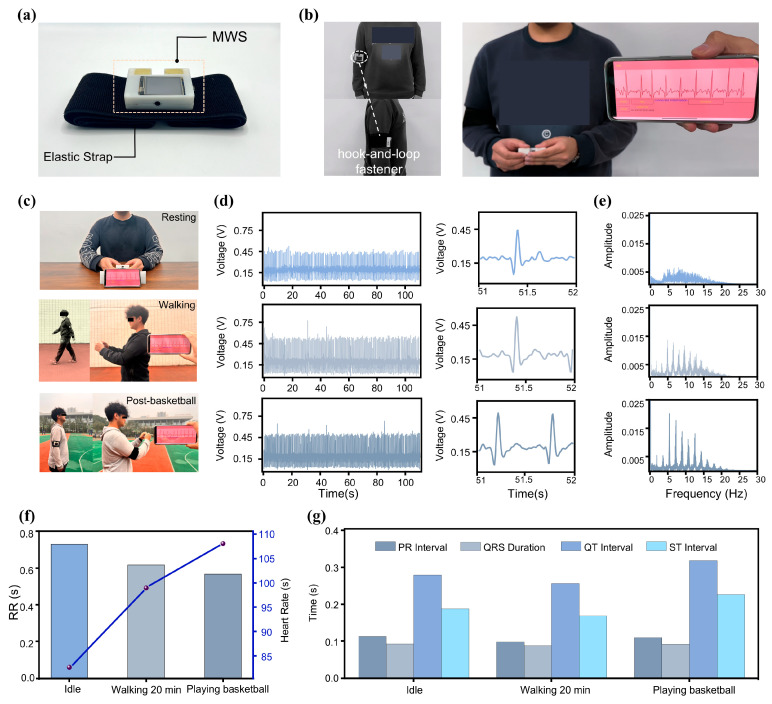
The MWS for ECG monitoring in various scenarios. (**a**) The MWS with band, its auxiliary fixation device, on the back. (**b**) MWS worn on the arm of Subject F. (**c**) Subject using the MWS for ECG monitoring in a different state. (**d**) Waveforms of ECG signals monitored in the three scenarios. (**e**) Frequency spectra (0 Hz–30 Hz) corresponding to the three conditions. (**f**,**g**) Comparison of ECG signal features in the three scenarios.

The MWS features a mechanical fastening mechanism on its backside (Figure 4a), enabling secure attachment to elastic bands for convenient and stable placement on the user’s arm (Figure 4b). When the user needs to perform an ECG signal measurement, the device can be effortlessly detached for ECG monitoring (Video S3 in the ESM). This unconstrained measurement strategy ensures comfort and wearability across diverse scenarios without disrupting daily activities, thereby significantly reducing the barrier to routine ECG monitoring. The design thoughtfully balances robust fixation during wear with seamless detachment for measurement, reflecting a well-integrated approach to practical usability and technical performance in wearable health monitoring systems.

To validate the practical applicability of the MWS across various scenarios, we tested ECG waveforms from the same individual during three distinct conditions: rest, walking, and post-basketball game (Figure 4c). For each condition, continuous ECG signal recordings lasting 110 s were acquired, and the resulting data, along with the detailed waveforms, are presented in Figure 4d. The MWS consistently captured high-quality ECG signals across all conditions. Notably, the ECG waveforms exhibited systematic changes corresponding to increasing physical activity levels. As exercise intensity increased, waveform cycles became shorter and heart rate (HR) accelerated. Fourier transform frequency spectrum (Figure 4e) revealed elevated overall autonomic nervous system activity during exercise, as reflected by increased total spectral power. Post-exercise measurements showed pronounced sympathetic activation, evidenced by enhanced low-frequency components, while elevated vagal tone and respiratory rates contributed to markedly increased power in the 5–20 Hz range [50]. These spectral changes were especially prominent when comparing the resting and post-basketball states.

To obtain more detailed information, we conducted an in-depth time-domain analysis of the subject’s ECG signal. As shown in Figure 4f, the HR and RR intervals under the three activity conditions were compared. With increasing exercise intensity, the duration of RR intervals decreased, resulting in a marked elevation in HR [51]. Figure 4g presents a comparison of additional characteristic parameters, all of which were extracted from the ECG signal. These results indicate that the MWS possesses strong potential for comprehensive and accurate unconstrained ECG monitoring across a variety of scenarios. Its high portability, combined with excellent performance in ECG signal acquisition, transmission, storage, and analysis, positions it as a promising tool for advancing portable physiological signal monitoring technologies.

### 3.5. Construction of Exercise Intensity Evaluation Model Based on ECG Features

**Figure 5:**
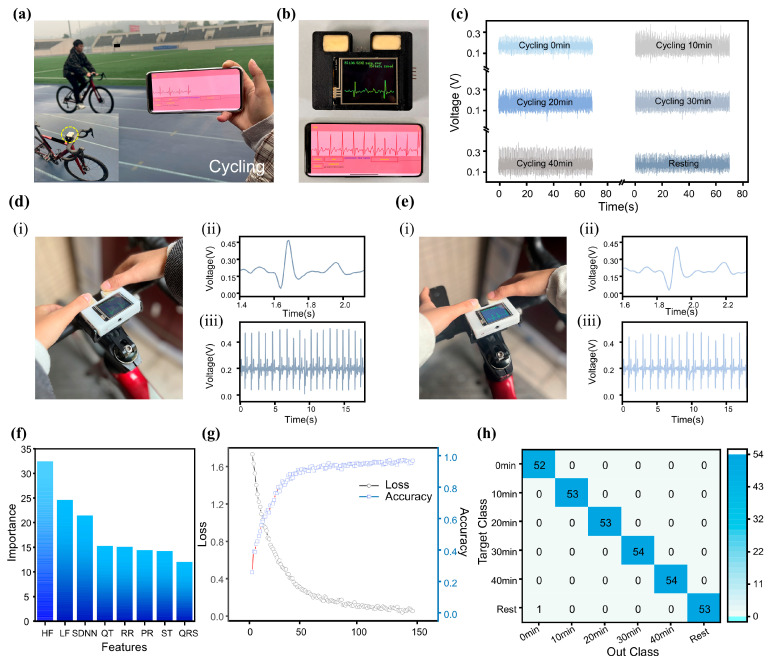
Model for motion intensity classification using the MWS. (**a**) Subject using the MWS for ECG monitoring during cycling. (**b**) Real-time ECG waveform display of the MWS on the screen and the smartphone application (APP). (**c**) ECG monitoring of Subject E during cycling at different time points. (**d**,**e**) Sweat resistance testing of the FBE. (**f**) Contribution analysis of ECG signal features used for model training. (**g**) Changes in classification accuracy and loss metric. (**h**) Confusion matrix comparing predicted and true labels after 146 training epochs.

Beyond wearable applications on the arm, the MWS can also be mounted on bicycle handlebars for exercise monitoring (Figure 5a). During slow cycling (Video S4 in the ESM), the MWS successfully captured ECG waveforms (Figure 5b). Although minor fluctuations caused by motion and muscle activity were present, all key ECG features remained identifiable. To further evaluate the system’s reliability during exercise, a subject performed continuous cycling for 40 min, with ECG signal (t=70 s) recordings taken every 10 min during rest intervals (single-cycle waveform comparison in Figure S9 in the ESM). As shown in Figure 5c, the results confirmed that ECG characteristics remained discernible throughout the test. Another potential challenge during exercise is the effect of sweat. Fortunately, the FBE’s protein and starch components possess strong moisture-absorbing capabilities, effectively reducing sweat-induced interference at the finger-electrode interface. Moreover, since the electrode relies on ion conduction from the internal electrolyte rather than a direct skin-electrode current path, signal acquisition remains stable even in the presence of sweat. Comparative tests were conducted on Subject E before (Figure 5d) and after sweat accumulation (Figure 5e). The results showed only minor variations in signal amplitude (≤0.50 mV) while maintaining complete ECG waveform integrity, demonstrating the strong potential of the MWS for exercise-related ECG monitoring applications.

Traditional wearable devices estimate exercise intensity based solely on single parameters (e.g., heart rate), which often results in limited accuracy and inherent constraints. Due to the complex nonlinear relationships among various ECG-derived features, nonlinear models, such as neural networks, are commonly employed in ECG signal analysis [52,53,54,55]. In this study, we developed a classification model based on an FCNN to assess exercise intensity. The model was trained using ECG waveforms collected during cycling sessions of varying durations (Figure 5c), simulating different levels of physical exertion. In contrast to time-series-sensitive convolutional neural network (CNN), the FCNN-based approach requires prior extraction of relevant ECG features (Figure S10 in the ESM) [56,57], enabling a simpler model architecture with reduced computational demands. The model’s input features include five time-domain ECG waveform characteristics (RR, PR, ST, QRS, and QT intervals) along with HRV parameters: standard deviation of normal-to-normal intervals (SDNN), high-frequency power (HF), and low-frequency power (LF). Detailed definitions and calculation methods are provided in Table S3 in the ESM. The ECG signal feature extraction process (Figure S11 in the ESM) incorporates first-order and second-order differentiation of the signal. Wave peaks are detected based on zero-crossing points in the first derivative, while wave polarity is determined using the extrema of the second derivative. The R-wave localization is achieved by identifying the point of maximum amplitude in each cycle, which then serves as a reference for locating other characteristic waves. This combined method offers computational efficiency while maintaining robust and accurate feature extraction, making it well-suited for integration with the FCNN algorithm. The resulting system enables accurate classification of exercise intensity with low computational overhead, supporting its implementation in resource-constrained wearable platforms. The model’s performance highlights the practicality of using simplified neural network architectures for physiological signal analysis in real-world wearable applications.

To prevent overfitting prior to and during training, the following mechanisms were implemented: 1. Dropout layers with a rate of *p* = 0.3 were incorporated into the neural network to constrain model complexity and reduce the risk of overfitting. 2. Training was halted if the validation loss failed to improve for 10 consecutive epochs. This strategy effectively mitigated overfitting on the training set while also enhancing computational efficiency, terminating training 54 epochs early, corresponding to a 27% reduction in training time, and optimizing the use of computational resources. After 146 training epochs, feature importance analysis revealed that among the extracted ECG characteristics, the HRV parameters contributed most significantly to the classification model’s performance (Figure 5f). This finding is consistent with established physiological understanding: during prolonged exercise, heart rate typically undergoes an initial acceleration followed by gradual stabilization [58,59], diminishing the relative utility of RR intervals for accurately assessing exercise intensity over extended periods. Following the completion of model training, classification testing was conducted using varying exercise durations to simulate different levels of exercise intensity. The classification process is detailed in Figure S12 in the ESM. All features extracted from the ECG signal sequences were input into the model’s input layer, and the final classification results were accurately produced after processing through two hidden layers. Evaluation results demonstrated that the model achieved a high classification accuracy of 98% after 146 training epochs (Figure 5g). Meanwhile, the confusion matrix of its output (Figure 5h) also demonstrates the model’s excellent classification performance. These results fully validate the outstanding capability of MWS in ECG monitoring and exercise intensity assessment.

### 3.6. Performance Comparison Between MWS and Commercial ECG Monitoring Devices

To further validate the innovativeness and advantages of the MWS, we conducted a comprehensive survey of several advanced portable ECG monitoring devices currently available on the market. A comparative analysis was carried out between the MWS and these commercial devices based on key metrics, including size, weight, and cost [60]. The detailed comparison results are presented in Table 1.

While current portable ECG monitoring systems on the market offer relatively high overall performance, there is still room for optimization in aspects such as size, cost, and functional integration.

As shown in the comparative data table, although the MWS does not exhibit overwhelming superiority in any single metric, it consistently performs within the upper-middle range across all key indicators. Notably, in terms of size and weight, two critical factors influencing portability, the MWS ranks second only to Kardia Mobile, demonstrating excellent compactness. Moreover, while most commercially available portable devices remain relatively expensive, the MWS offers a clear cost advantage, underscoring its competitive value. It is also important to note that few existing devices provide real-time ECG waveform display functionality. Although some products, such as Kardia Mobile, support data transmission to smartphones for visualization via Bluetooth, this approach introduces operational dependencies and may limit usability in certain scenarios.

In summary, the MWS presents a well-balanced combination of size, weight, cost, and standalone portability, indicating strong potential for broad application in portable ECG monitoring.

## 4. Conclusions

This work proposes a miniaturized, unconstrained ECG monitoring system that utilizes a low-cost, easily fabricated FBE with high moldability. The system is designed to efficiently monitor individual physiological signals and provide an effective means of assessing human exercise intensity. The proposed miniaturized system features a highly compact form factor, measuring just 65 × 52 × 12 mm^3^, and a total mass of only 69 g. Its small size and low weight enable unobtrusive wearability on the user’s forearm or straightforward mounting to a bicycle frame. Despite these constraints, the device is capable of reliably detecting and delineating the characteristic fiducial points of the ECG waveform under a variety of operating conditions. The integrated efficient filtering circuitry ensures high-quality ECG signal acquisition while maintaining signal integrity even in the presence of powerline interference. Regarding the electrodes, the FBE demonstrates a high contact signal-to-noise ratio of 33 dB and permits arbitrary reshaping without the need for mold prefabrication. Consequently, it can be readily integrated into diverse embedded platforms, thereby substantially reducing the overall cost of physiological signal monitoring. The FBE exhibits both extremely low production cost and excellent conductive ability, showing lower contact impedance with human skin in low-frequency ranges compared to commercial Ag/AgCl gel electrodes. When properly stored in humid conditions, the FBE maintains reliable ECG monitoring performance after one month of use, demonstrating high reusability. Based on the high-quality ECG signal acquired, we established a fully connected neural network-based physical activity intensity classification model that achieved 98% accuracy after 146 training epochs, indicating the outstanding potential of the MWS in human activity intensity assessment. This study represents a new era of cost-effective and unconstrained signal monitoring, serving as an effective alternative to traditional constrained ECG monitoring devices, particularly suitable for fitness enthusiasts. The system’s high portability and intelligent analysis capabilities significantly enhance user convenience while promoting the development of noninvasive and more comfortable remote health monitoring platforms.

## Figures and Tables

**Table 1:** Comparative analysis of performance metrics for different ECG monitoring devices.

Product	Kardia Mobile	Omron HeartScan	REKA E100	AfibAlert	MWS
Dimensions (mm^3^)	82×32×3.5	121×67×24	70×81×17.4	149×69×28	65×52×12
Weight (g)	18	130	105	184	63
Price	79(USD)	42,500(EUR)		249(USD)	50(CNY)
On-device Display	Requires smartphone	YES	NO	NO	YES

## Data Availability

The data that support the findings of this study are available from the corresponding author upon reasonable request.

## Supplementary Materials

The following supporting information can be downloaded at https://www.mdpi.com/article/10.3390/s25134213/s1, Figure S1. A detailed multi-angle view of the MWS. Figure S2. Experimental setup for measuring the relationship between the FBE resistance and humidity. Figure S3. Electrochemical analysis of the FBE in the frequency range of 20 Hz to 15 kHz with a cross-sectional area of *S* = 1 cm^2^ and a length of *L* = 2.5 cm. Figure S4. Resistivity variation of the FBE over ten days. Figure S5. Notch filter circuit for power frequency interference. Figure S6. Software flowchart of the microcontroller within the MWS. Figure S7. Accuracy validation of the MWS’s ECG monitoring using a standard multi-parameter patient monitor. Figure S8. Waveform of ECG signal measurement using the FBE over 30 days. Figure S9. ECG signals of the subject during cycling at different time points. Figure S10. Features of ECG signals used for training the classification model. Figure S11. ECG waveform and its first and second derivatives. Figure S12. Structure of the classification model for exercise intensity recognition. Table S1. Comparison of costs and ECG signal detection methods between MWS and other works. Table S2. Information on the instruments used in the experiments of this study. Table S3. Description and formulas of the feature values of ECG signals used for the exercise intensity classification model. Video S1 to S4.

## References

[1] Aziz S., Ahmed S., Alouini M.-S. (2021). ECG-based machine-learning algorithms for heartbeat classification. Sci. Rep..

[2] Barrett P.M., Komatireddy R., Haaser S., Topol S., Sheard J., Encinas J., Fought A.J., Topol E.J. (2014). Comparison of 24-hour Holter Monitoring with 14-day Novel Adhesive Patch Electrocardiographic Monitoring. Am. J. Med..

[3] Herman R., Kisova T., Belmonte M., Wilgenhof A., Toth G., Demolder A., Rafajdus A., Meyers H.P., Smith S.W., Bartunek J. (2025). Artificial Intelligence-Powered Electrocardiogram Detecting Culprit Vessel Blood Flow Abnormality: AI-ECG TIMI Study Design and Rationale. J. Soc. Cardiovasc. Angiogr. Interv..

[4] Nakamura T., Sasano T. (2025). Emerging Potential and Challenges of AI-Based ECG Analysis in Clinical Medicine. JACC Asia.

[5] Yang Y., Tian X.Y., Sun P.F., Zhao X.L., Hu J.T., Pan B. (2024). Electrocardiographic abnormalities in patients with microtia. Sci. Rep..

[6] Yıldırım Ö., Pławiak P., Tan R.-S., Acharya U.R. (2018). Arrhythmia detection using deep convolutional neural network with long duration ECG signals. Comput. Biol. Med..

[7] Franklin D., Tzavelis A., Lee J.Y., Chung H.U., Trueb J., Arafa H., Kwak S.S., Huang I., Liu Y., Rathod M. (2023). Synchronized wearables for the detection of haemodynamic states via electrocardiography and multispectral photoplethysmography. Nat. Biomed. Eng..

[8] Khunte A., Sangha V., Oikonomou E.K., Dhingra L.S., Aminorroaya A., Mortazavi B.J., Coppi A., Brandt C.A., Krumholz H.M., Khera R. (2023). Detection of left ventricular systolic dysfunction from single-lead electrocardiography adapted for portable and wearable devices. npj Digit. Med..

[9] Kiranyaz S., Ince T., Gabbouj M. (2017). Personalized Monitoring and Advance Warning System for Cardiac Arrhythmias. Sci. Rep..

[10] Lin Z.X., Kireev D., Liu N., Gupta S., Lapiano J., Obaid S.N., Chen Z.Y., Akinwande D., Efimov I.R. (2023). Graphene Biointerface for Cardiac Arrhythmia Diagnosis and Treatment. Adv. Mater..

[11] Muthukumar K.A., Nandi D., Ranjan P., Ramachandran K., Pj S., Ghosh A., M A., Radhakrishnan A., Dhandapani V.E., Janardhanan R. (2025). Integrating electrocardiogram and fundus images for early detection of cardiovascular diseases. Sci. Rep..

[12] Tricoli A., Nasiri N., De S.Y. (2017). Wearable and Miniaturized Sensor Technologies for Personalized and Preventive Medicine. Adv. Funct. Mater..

[13] Wu W.T., Li L.L., Li Z.X., Sun J.Z., Wang L.L. (2023). Extensible Integrated System for Real-Time Monitoring of Cardiovascular Physiological Signals and Limb Health. Adv. Mater..

[14] World Health Organization (2024). World Health Statistics 2024: Monitoring Health for the SDGs, Sustainable Development Goals. https://www.who.int/publications/i/item/9789240094703.

[15] Lindstrom M., Decleene N., Dorsey H., Fuster V., Johnson C.O., Legrand K.E., Mensah G.A., Razo C., Stark B., Varieur Turco J. (2022). Global Burden of Cardiovascular Diseases and Risks Collaboration, 1990–2021. J. Am. Coll. Cardiol..

[16] Roth G.A., Mensah G.A., Johnson C.O., Addolorato G., Ammirati E., Baddour L.M., Barengo N.C., Beaton A.Z., Benjamin E.J., Benziger C.P. (2020). Global Burden of Cardiovascular Diseases and Risk Factors, 1990–2019: Update from the GBD 2019 Study. J. Am. Coll. Cardiol..

[17] Wang H.D., Naghavi M., Allen C., Barber R.M., Bhutta Z.A., Carter A., Casey D.C., Charlson F.J., Chen A.Z., Coates M.M. (2016). Global, regional, and national life expectancy, all-cause mortality, and cause-specific mortality for 249 causes of death, 1980–2015: A systematic analysis for the Global Burden of Disease Study 2015. Lancet.

[18] Driscoll N., Erickson B., Murphy B.B., Richardson A.G., Robbins G., Apollo N.V., Mentzelopoulos G., Mathis T., Hantanasirisakul K., Bagga P. (2021). MXene-infused bioelectronic interfaces for multiscale electrophysiology and stimulation. Sci. Transl. Med..

[19] O’neill S.J.K., Huang Z.H., Ahmed M.H., Boys A.J., Velasco-Bosom S., Li J.X., Owens R.M., Mccune J.A., Malliaras G.G., Scherman O.A. (2023). Tissue-Mimetic Supramolecular Polymer Networks for Bioelectronics. Adv. Mater..

[20] Wang S., Nie Y., Zhu H., Xu Y., Cao S., Zhang J., Li Y., Wang J., Ning X., Kong D. (2022). Intrinsically stretchable electronics with ultrahigh deformability to monitor dynamically moving organs. Sci. Adv..

[21] Hossain M.M., Li B.M., Sennik B., Jur J.S., Bradford P.D. (2022). Adhesive free, conformable and washable carbon nanotube fabric electrodes for biosensing. npj Flex. Electron..

[22] Lee S., Ho D.H., Jekal J., Cho S.Y., Choi Y.J., Oh S., Choi Y.Y., Lee T., Jang K.-I., Cho J.H. (2024). Fabric-based lamina emergent MXene-based electrode for electrophysiological monitoring. Nat. Commun..

[23] Niu L., Shen Z., Wang Z., Qi L.Y., Niu H.T., Zhou H., Zhang C., Xu J., Fang J. (2024). Low-Contact Impedance Textile Electrode for Real-Time Detection of ECG Signals. ACS Appl. Mater..

[24] Kruse K., Sauerwein W., Lübben J., Dodel R. (2024). Smart technologies and textiles and their potential use and application in the care and support of elderly individuals: A systematic review. Rev. Adv. Mater. Sci..

[25] Villar R., Beltrame T., Hughson R.L. (2015). Validation of the Hexoskin wearable vest during lying, sitting, standing, and walking activities. Appl. Physiol. Nutr. Metab..

[26] Hashimoto Y., Sato R., Takagahara K., Ishihara T., Watanabe K., Togo H. (2022). Validation of Wearable Device Consisting of a Smart Shirt with Built-In Bioelectrodes and a Wireless Transmitter for Heart Rate Monitoring in Light to Moderate Physical Work. Sensors.

[27] Dong J.C., Wang D., Peng Y.D., Zhang C., Lai F.L., He G.J., Ma P.M., Dong W.F., Huang Y.P., Parkin I.P. (2022). Ultra-stretchable and superhydrophobic textile-based bioelectrodes for robust self-cleaning and personal health monitoring. Nano Energy.

[28] Chi Y.M., Jung T.P., Cauwenberghs G. (2010). Dry-Contact and Noncontact Biopotential Electrodes: Methodological Review. IEEE Rev. Biomed. Eng..

[29] Li T., Qi H.B., Zhao C.C., Li Z.M., Zhou W., Li G.J., Zhuo H., Zhai W. (2025). Robust skin-integrated conductive biogel for high-fidelity detection under mechanical stress. Nat. Commun..

[30] Wang F.C., Xue Y., Chen X.M., Zhang P., Shan L.J., Duan Q.F., Xing J.F., Lan Y., Lu B.Y., Liu J. (2024). 3D Printed Implantable Hydrogel Bioelectronics for Electrophysiological Monitoring and Electrical Modulation. Adv. Funct. Mater..

[31] Wang P., Lv Y., Duan J.L., Sun G.F., Meng C.Z., Li Y., Guo S.J., Zhang T. (2025). A thermally responsive phase-change hydrogel for skin-mountable multifunctional sensors. Nano Energy.

[32] Xu K., Wang L.M., Shan W.J., Gao K., Wang J.P., Zhong Q., Zhou W.L. (2024). Highly Stretchable and Self-Adhesive Wearable Biosensor Based on Nanozyme-Catalyzed Conductive Hydrogels. ACS Appl. Polym. Mater..

[33] Liu C.C., Wang Y.Y., Shi S.T., Zheng Y.B., Ye Z.W., Liao J.Q., Sun Q.F., Dang B.K., Shen X.P. (2024). Myelin Sheath-Inspired Hydrogel Electrode for Artificial Skin and Physiological Monitoring. ACS Nano.

[34] Le K., Sun X., Chen J.J., John J.V., Servati A., Heidari H., Khademhosseini A., Ko F., Jiang F., Servati P. (2023). Stretchable, self-healing, biocompatible, and durable ionogel for continuous wearable strain and physiological signal monitoring. Chem. Eng. J..

[35] Qiu J.K., Yu T.H., Zhang W.F., Zhao Z.H., Zhang Y., Ye G., Zhao Y., Du X.J., Liu X., Yang L. (2020). A Bioinspired, Durable, and Nondisposable Transparent Graphene Skin Electrode for Electrophysiological Signal Detection. Mater. Lett..

[36] Wang Q., Ling S.J., Liang X.P., Wang H.M., Lu H.J., Zhang Y.Y. (2019). Self-Healable Multifunctional Electronic Tattoos Based on Silk and Graphene. Adv. Funct. Mater..

[37] Joutsen A., Cömert A., Kaappa E., Vanhatalo K., Riistama J., Vehkaoja A., Eskola H. (2024). ECG signal quality in intermittent long-term dry electrode recordings with controlled motion artifacts. Sci. Rep..

[38] Zhang L., Kumar K.S., He H., Cai C.J., He X., Gao H.X., Yue S.Z., Li C.S., Seet R.C.-S., Ren H.L. (2020). Fully organic compliant dry electrodes self-adhesive to skin for long-term motion-robust epidermal biopotential monitoring. Nat. Commun..

[39] Mirbakht S.S., Golparvar A., Umar M., Kuzubasoglu B.A., Irani F.S., Yapici M.K. (2025). Highly Self-Adhesive and Biodegradable Silk Bioelectronics for All-In-One Imperceptible Long-Term Electrophysiological Biosignals Monitoring. Adv. Sci..

[40] Lu J., Li Q.M., Huang Q.Y., Li D., Jiao Y.D., Wang Y.Z., Li Y., Zou K.Y., Chen Z.L., Gu J.Y. (2025). A Highly Sensitive Surface Electrode for Electrophysiological Monitoring. Adv. Funct. Mater..

[41] Zheng Y., Li Y.K., Zhao Y., Lin X.H., Luo S.C., Wang Y., Li L.L., Teng C., Wang X.L., Xue G. (2023). Ultrathin and highly breathable electronic tattoo for sensing multiple signals imperceptibly on the skin. Nano Energy.

[42] Vikhe R., Masure S., Das M.K., Mishra A., Sreehari E., Kulhari U., Sahu B.D., Sharma L.N., Loganathan S., Kumar S. (2025). Fabrication, Characterization, and Clinical Assessment of Ultrathin Skin-Conformable Tattoo Electrodes for ECG Monitoring. ACS Appl. Electron. Mater..

[43] Gao J.W., Hu M.W., Sun H., Wang Y.Y., Wei Y., Li W.W., Zheng L., Xu M.Z., Lu Q.B., Liu Z.Y. (2025). Disposable and flexible smart electronic tapes for long-term biopotential monitoring. npj Flex. Electron..

[44] Chen J.X.M., Chen T.H., Zhang Y.X., Fang W.Q., Li W.E., Li T., Popovic M.R., Naguib H.E. (2024). Conductive Bio-based Hydrogel for Wearable Electrodes via Direct Ink Writing on Skin. Adv. Funct. Mater..

[45] Luo J.B., Sun C.Y., Chang B.Y., Zhang B., Li K., Li Y.G., Zhang Q.H., Wang H.Z., Hou C.Y. (2024). On-Skin Paintable Water-Resistant Biohydrogel for Wearable Bioelectronics. Adv. Funct. Mater..

[46] Zang J.B., An Q., Li B., Zhang Z.D., Gao L.B., Xue C.Y. (2025). A novel wearable device integrating ECG and PCG for cardiac health monitoring. Microsyst. Nanoeng..

[47] Takeshita T., Yoshida M., Takei Y., Ouchi A., Hinoki A., Uchida H., Kobayashi T. (2022). Development of wearable multi-lead ECG measurement device using cubic flocked electrode. Sci. Rep..

[48] Wang W.T., Lu L.S., Ma H., Li Z.H., Lu X.Y., Xie Y.X. (2024). Self-template manufacturing of on-skin electrodes with 3D multi-channel structure for standard 3-limb-lead ECG suit. Microsyst. Nanoeng..

[49] Mauritzen C.a.M., Stewart P. (1963). Disulphide-Sulphydryl Exchange in Dough. Nature.

[50] Talavera J.R., Mendoza E.A.S., Dávila N.M., Supo E. Implementation of a real-time 60 Hz interference cancellation algorithm for ECG signals based on ARM cortex M4 and ADS1298. Proceedings of the 2017 IEEE XXIV International Conference on Electronics, Electrical Engineering and Computing (INTERCON).

[51] Shaffer F., Ginsberg J.P. (2017). An Overview of Heart Rate Variability Metrics and Norms. Front. Public Health.

[52] Hong S., Zhou Y., Shang J., Xiao C., Sun J. (2020). Opportunities and challenges of deep learning methods for electrocardiogram data: A systematic review. Comput. Biol. Med..

[53] Kwon J.-M., Kim K.-H., Jeon K.-H., Kim H.M., Kim M.J., Lim S.-M., Song P.S., Park J., Choi R.K., Oh B.-H. (2019). Development and Validation of Deep-Learning Algorithm for Electrocardiography-Based Heart Failure Identification. Korean Circ. J..

[54] Ribeiro A.H., Ribeiro M.H., Paixao G.M.M., Oliveira D.M., Gomes P.R., Canazart J.A., Ferreira M.P.S., Andersson C.R., Macfarlane P.W., Meira W. (2020). Automatic diagnosis of the 12-lead ECG using a deep neural network. Nat. Commun..

[55] Toma T.I., Choi S. (2022). A Parallel Cross Convolutional Recurrent Neural Network for Automatic Imbalanced ECG Arrhythmia Detection with Continuous Wavelet Transform. Sensors.

[56] Han D., Qi H., Hou D., Wang S., Kong J., Xu X., Wang C. (2025). Dynamic detection mechanism model of acoustic emission for high-speed train axle box bearings with local defects. Mech. Syst. Signal Process.

[57] Han D., Qi H., Wang S., Hou D., Wang C. (2024). Adaptive step size forward-backward pursuit and acoustic emission-based health state assessment of high-speed train bearings. Struct. Health Monit..

[58] Borresen J., Lambert M.I. (2007). Changes in heart rate recovery in response to acute changes in training load. Eur. J. Appl. Physiol..

[59] Wan H.-Y., Bunsawat K., Amann M. (2023). Autonomic cardiovascular control during exercise. Am. J. Physiol-Heart C.

[60] Bansal A., Joshi R. (2018). Portable out-of-hospital electrocardiography: A review of current technologies. J. Arrhythmia.

