# Supplementary material for: Flexible Bioelectrodes-Integrated Miniaturized System for Unconstrained ECG Monitoring
Source: Sensors (Basel). 2025 Jul 6;25(13):4213. doi: 10.3390/s25134213 (PMC12252470; doi:10.3390/s25134213)
Supplement: Supplementary file 1 [file sensors-25-04213-s001.zip › sensors-3695581-supplementary.pdf]

Electronic Supplementary Material

Flexible Bioelectrodes-Integrated Miniaturized System for Un-constrained ECG Monitoring

Yaoliang Zhan [1], Xue Wang [1,2,*] and Jin Yang [3,*]

[1] Chongqing Key Laboratory of Photo-Electronic Functional Materials and Laser Technology, College of Physics and Electronic Engineering, Chongqing Normal University, Chongqing 401331, China

[2] Chongqing Municipal Key Laboratory of Photo-Electronic Materials And Engineering of Higher Education, College of Physics and Electronic Engineering, Chongqing Normal University, Chongqing 401331, China

[3] Department of Optoelectronic Engineering, Key Laboratory of Optoelectronic Technology and Systems, Ministry of Education, Chongqing University, Chongqing 400044, China

The WORD file includes:

Figure S1 to S12
Table S1 to S3.

Other Supplementary Material for this manuscript includes the following:

Video S1 to S4

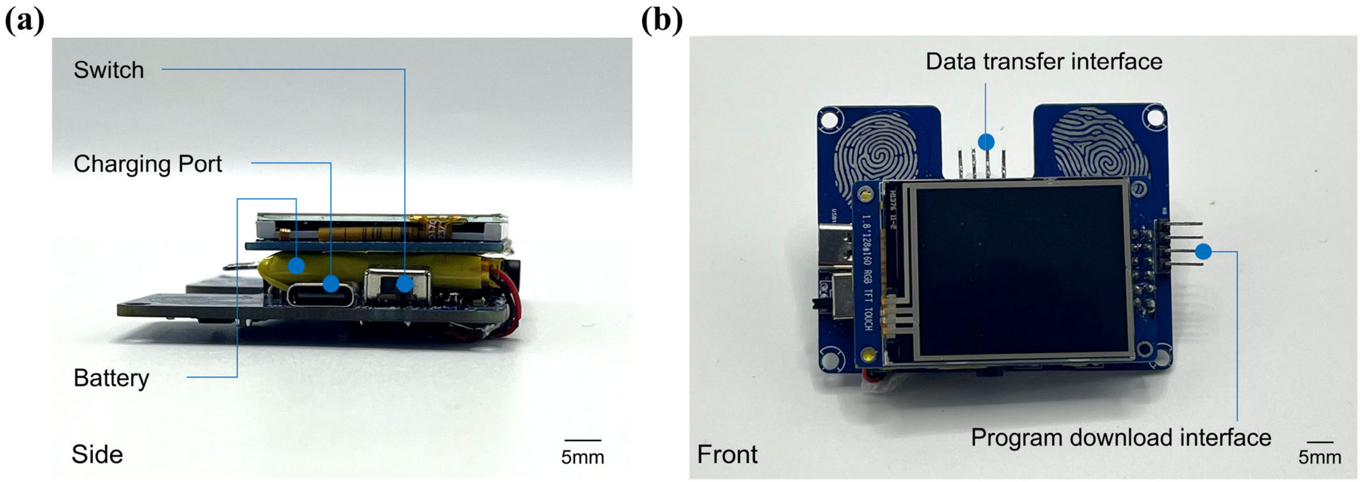

Figure S1. A detailed multi-angle view of the MWS. (a) 3D view of the MWS. (b) Front view of the MWS.

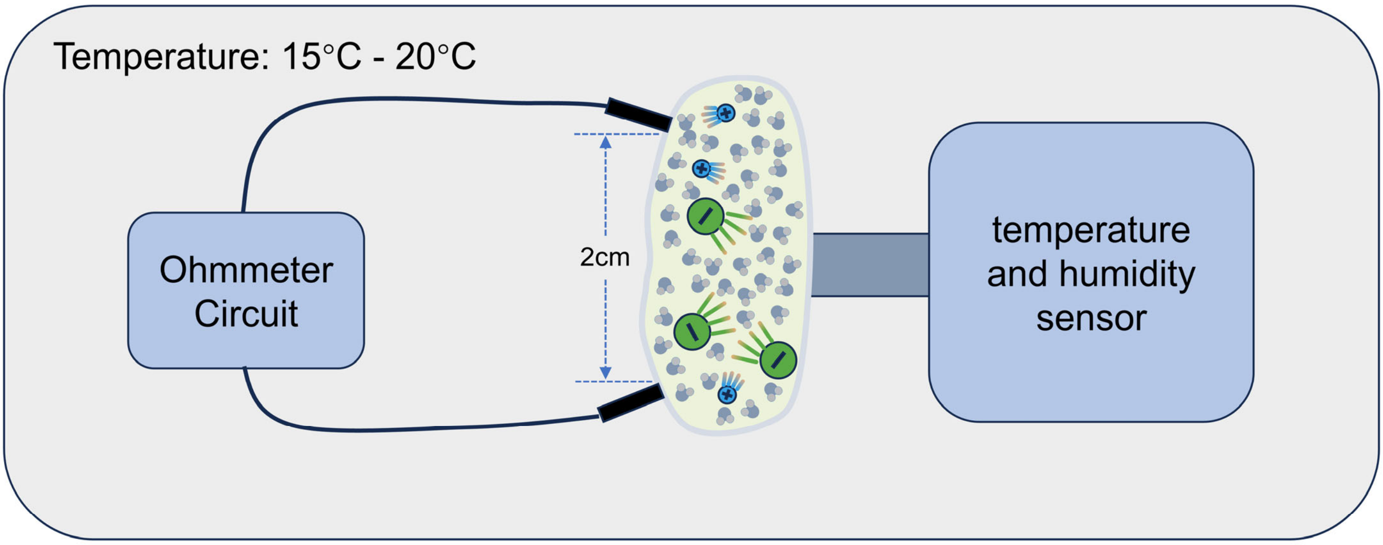

Figure S2. Experimental setup for measuring the relationship between the FBE resistance and humidity.

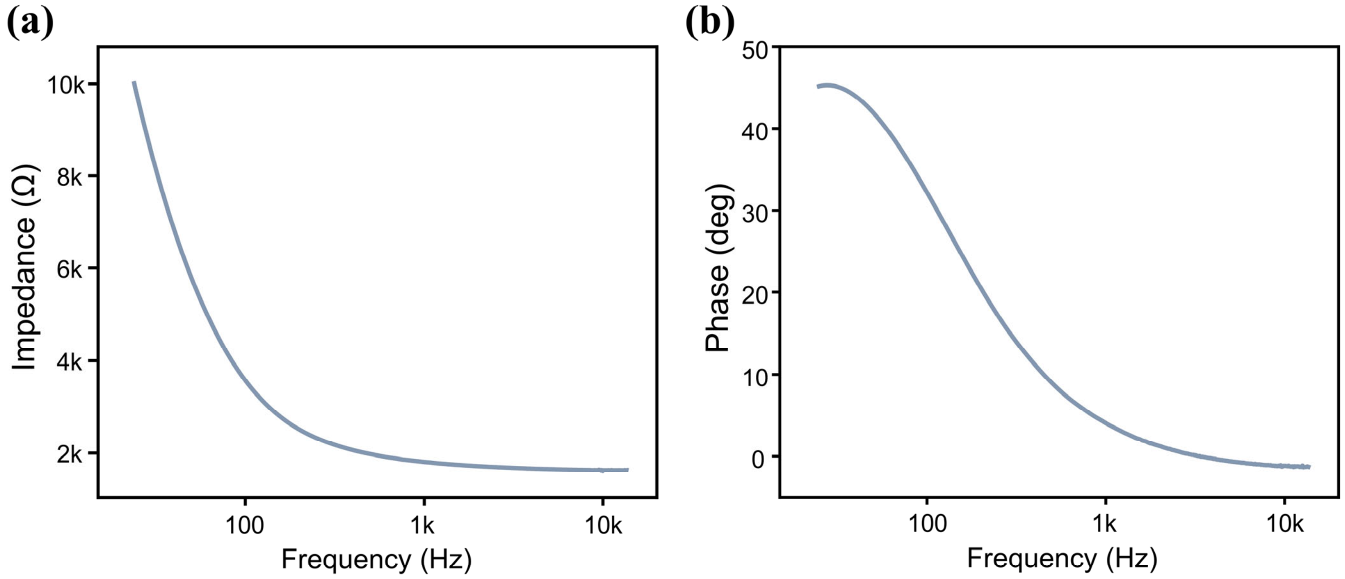

Figure S3. Electrochemical analysis of the FBE in the frequency range of 20 Hz to 15 kHz with a cross-sectional area of $S = 1$ cm² and a length of $L = 2.5$ cm. (a) Electrochemical impedance. (b) Electrochemical phase.

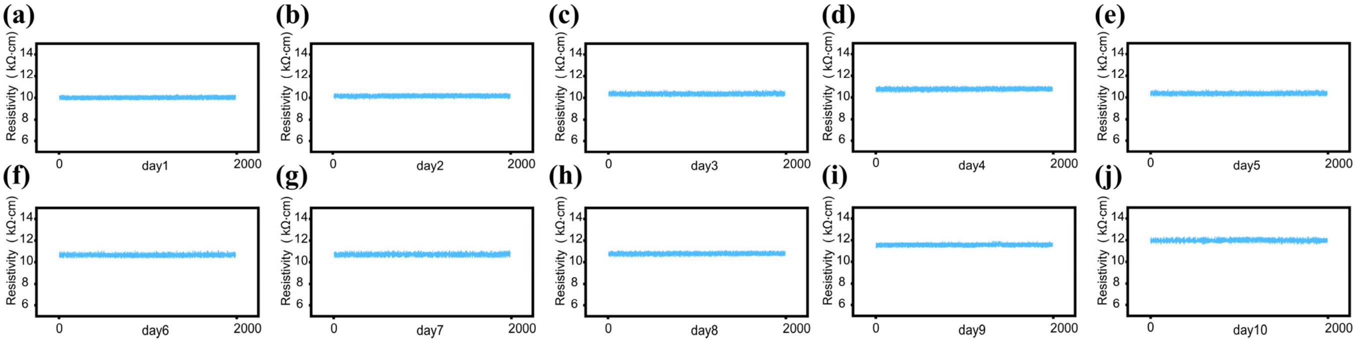

Figure S4. Resistivity variation of the FBE over ten days. (a)-(j) corresponding to days 1-10.

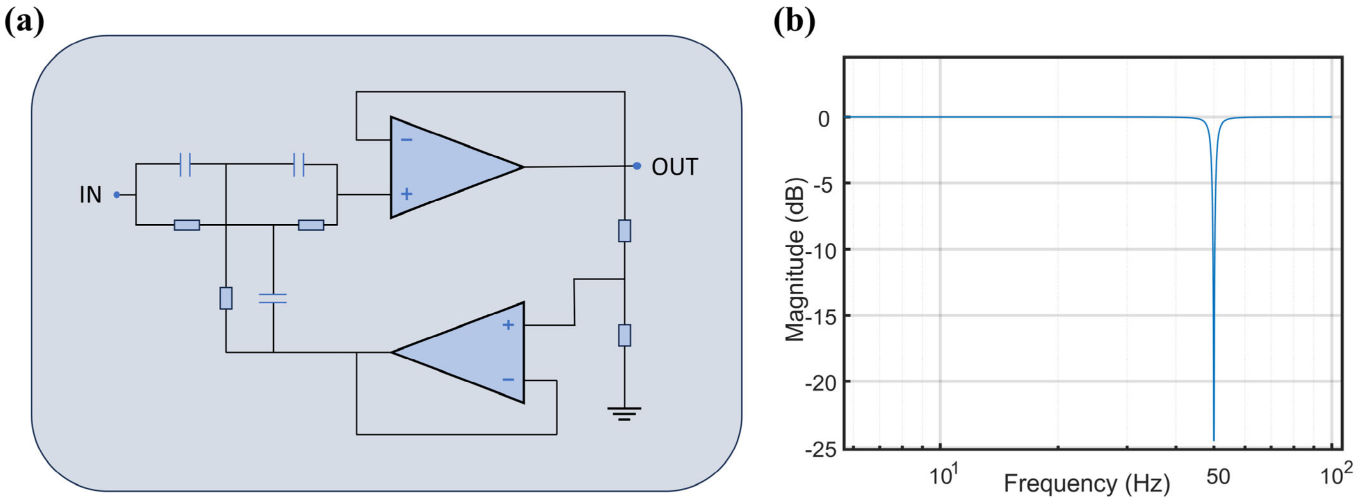

Figure S5. Notch filter circuit for power frequency interference. (a) Topology of the notch filter. (b) Frequency response of the notch filter.

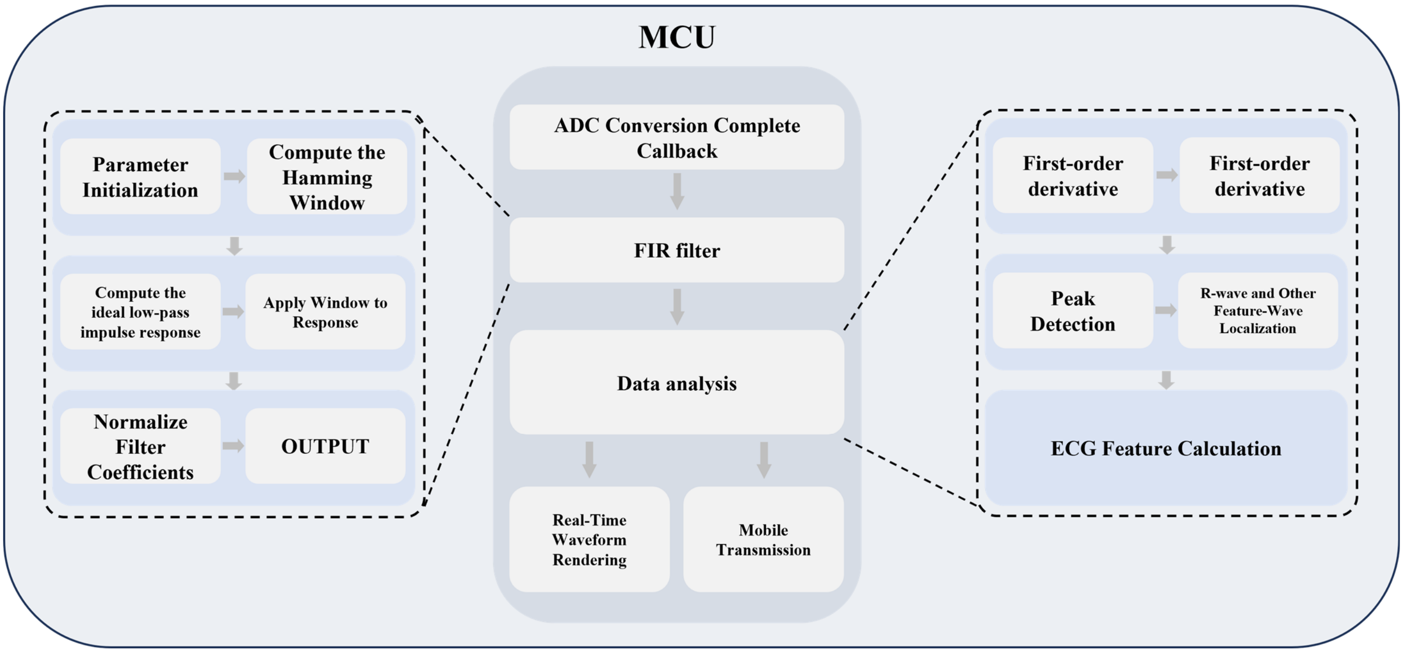

Figure S6. Software flowchart of the microcontroller within the MWS.

.

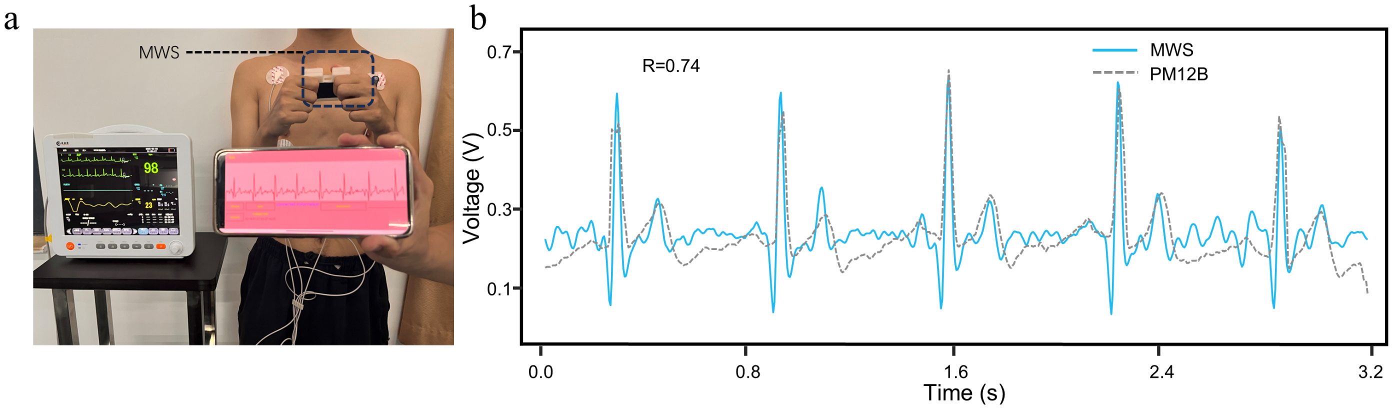

Figure S7. Accuracy validation of the MWS's ECG monitoring using a standard multi-parameter patient monitor. (a) Experimental Protocol. (b) Comparison of Resultant Waveforms.

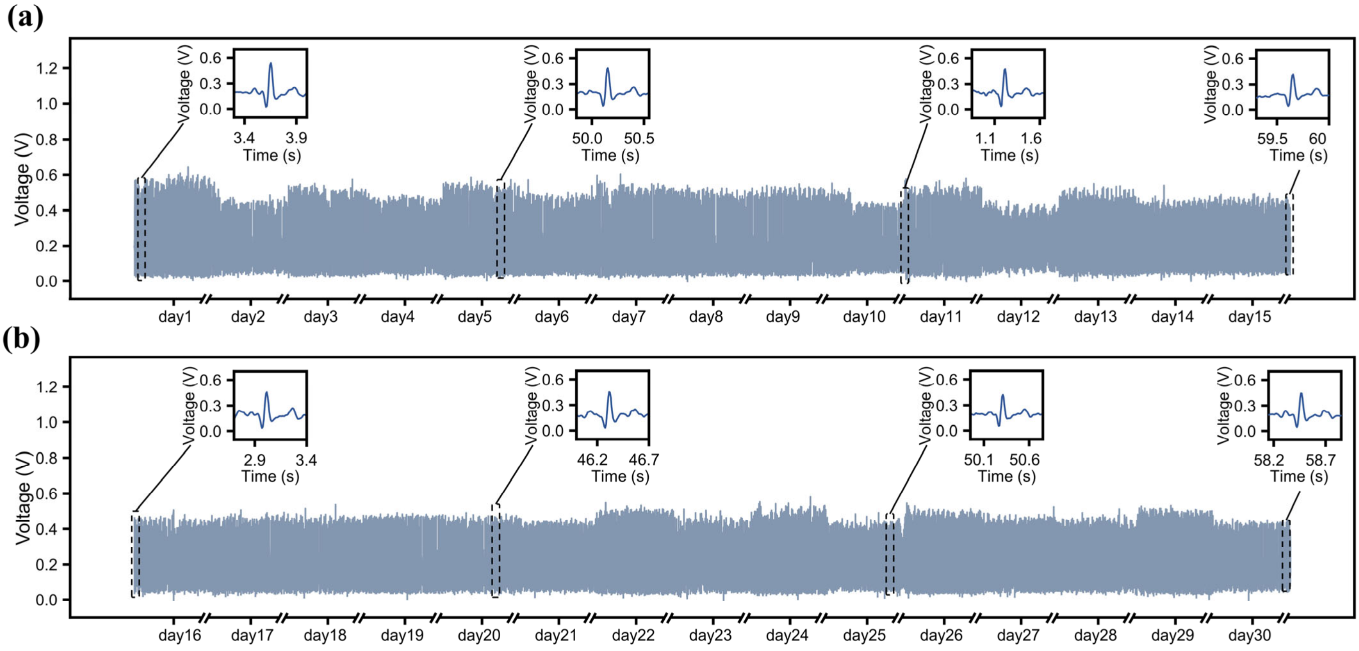

Figure S8. Waveform of ECG signal measurement using the FBE over 30 days.

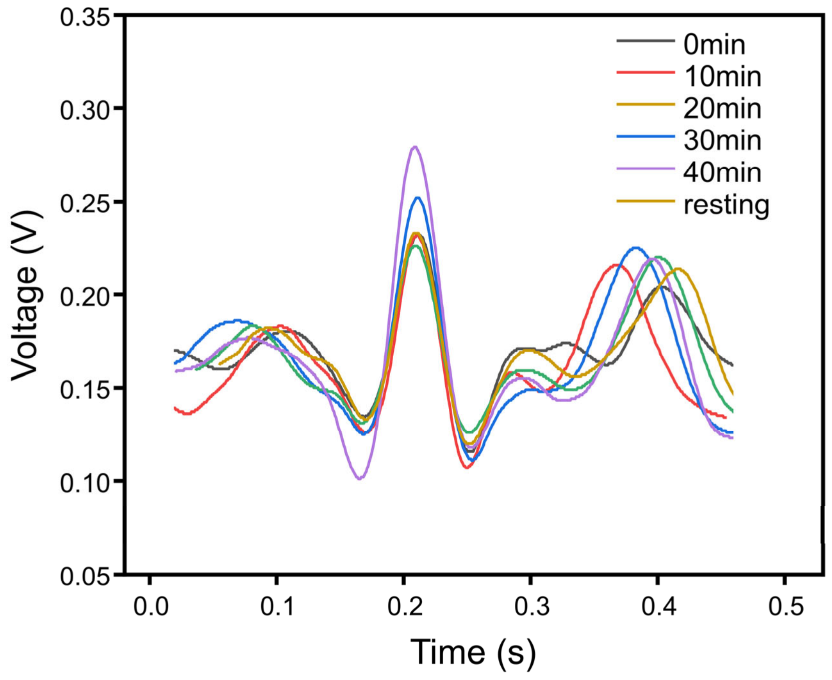

Figure S9. ECG signals of the subject during cycling at different time points.

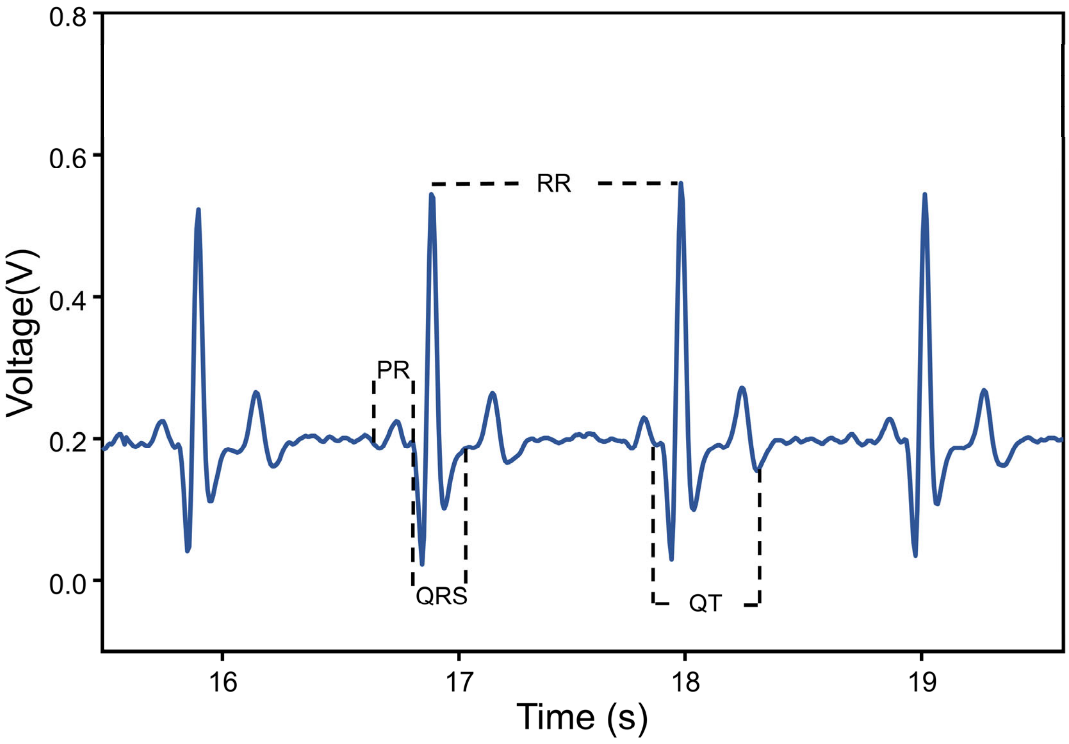

Figure S10. Features of ECG signals used for training the classification model.

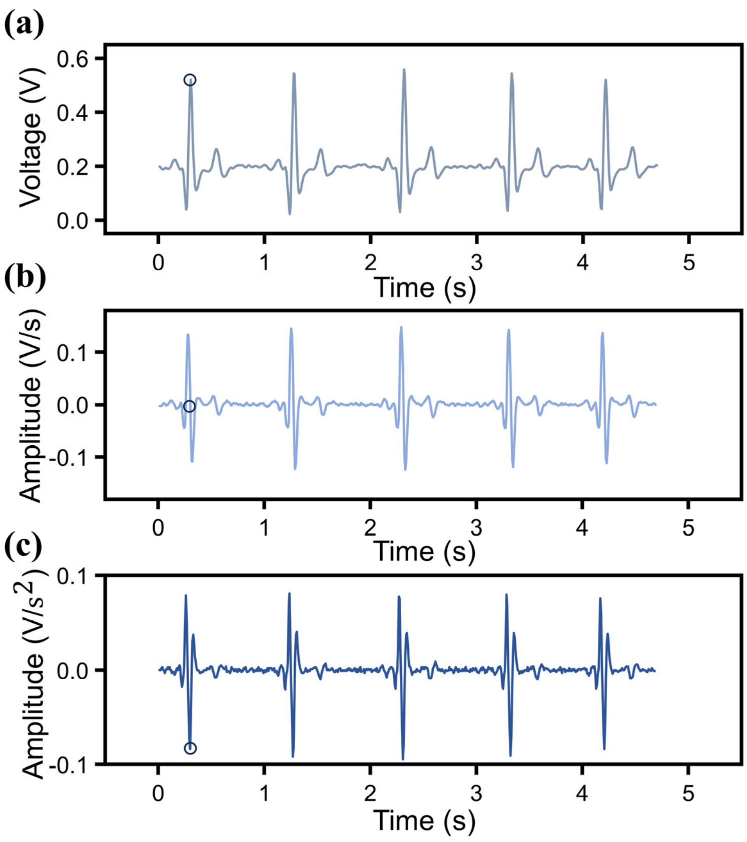

Figure S11. ECG waveform and its first and second derivatives. (a) Waveform of the ECG signal. (b) It's the first-order derivative. (c) Second-order derivative for feature wave capture.

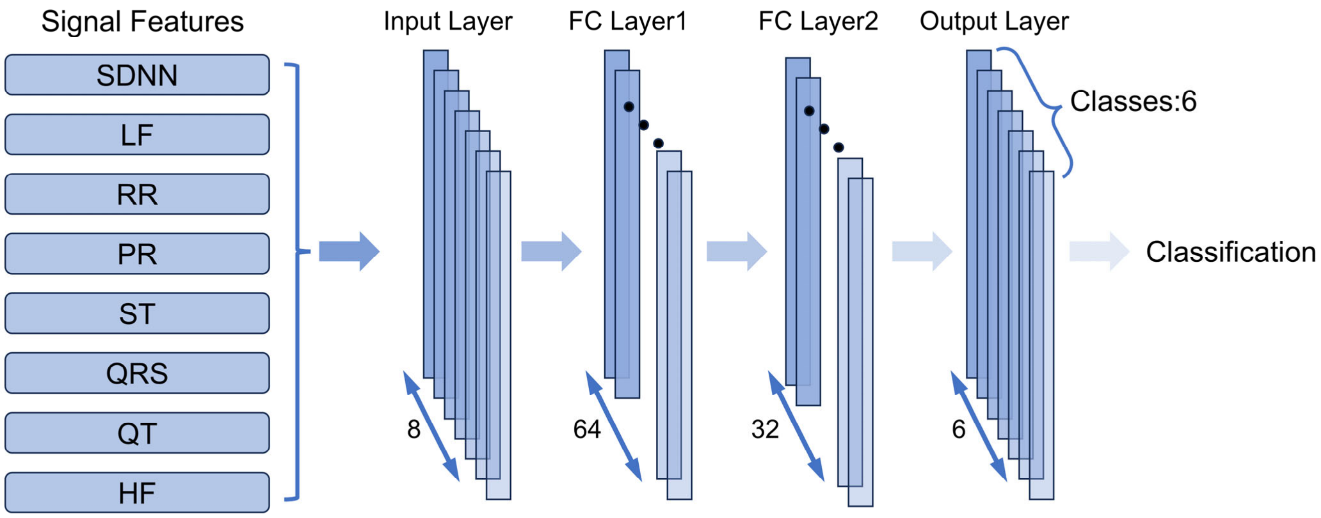

Figure S12. Structure of the classification model for exercise intensity recognition.

Table S1. Comparison of costs and ECG signal detection methods between MWS and other works.

Systems	Raw material	Cost (CNY)	Monitoring method
Ref [43]	Ag/AgCl	1-5 per	Constrained
Ref [44]	Ag-plated fibers	500-200 per kg	Constrained
	Urethane foam	50-200 per m^2	
Ref [45]	Ag-coated Fibers (AgCF)	1000-3000 per kg	Constrained
	Polyvinyl Alcohol (PVA)	50-200 per kg	
	Silicone elastomer	100-500 per kg	
This work	Industrial Salt	1.80-7.50per kg	Unconstrained
	Tartaric Acid	100-300 per kg	
	Vegetable Oil	5-20 per L	

Table S2. Information on the instruments used in the experiments of this study.

Measurement	Instrument	Model
Impedance<br>Phase	Impedance Analyzer	Wk6500, Guangzhou Junda Instruments & Meters
Temperature<br>Humidity	Temperature-Humidity Sensor	AHT25, Guangzhou Aosong Electronic
Resistance	Voltage Divider Circuit	custom-made PCB

Table S3. Description and formulas of the feature values of ECG signals used for the exercise intensity classification model.

Symbol	Description	Formula	Notes
HRV	Heart Rate Variability	$\sqrt{\frac{1}{N-1}\sum_{i=1}^{N}\left(T_{RR_i}-\mu\right)^2}$	μ: Mean R-R interval
LF	Low Frequency	$\sum_{f\in[0.04,0.15)} P(f)$	Linked to sympathetic activity
HF	High Frequency	$\sum_{f\in(0.15,0.4]} P(f)$	Linked to parasympathetic activity
$P(f)$	Power Spectrum of Frequency	$\frac{\left\|FFT\{x(t)\}\right\|^2}{L}$	Assessment of autonomic nervous system activity
T_{RR_i}	R-R interval	$t_{R_{i+1}}-t_{R_i}$	Heart rate-related parameter
$x(t)$	Interpolated RR interval time series	Interpolated from T_{RR_i}	Reflects heart rate trends over time
